# Phase Angle as a Predictor of Walking Independence in Patients Undergoing Stroke Rehabilitation: A Preliminary Study

**DOI:** 10.1298/ptr.25-E10354

**Published:** 2025-09-05

**Authors:** Naoya IKEDA, Yasuhiro MINAMIMURA

**Affiliations:** Department of Rehabilitation, Saiseikai Kibi Hospital, Japan

**Keywords:** Stroke, Phase angle, Muscle quality, Walking

## Abstract

**Objectives:**

This study examined the relationship between phase angle (PhA), an indicator of muscle quality, and independent walking in stroke patients. The objective was to determine the predictive value of a PhA at admission for walking independence at discharge.

**Methods:**

This study included 220 stroke patients (121 males, 99 females), categorized based on their functional independence measure (FIM) mobility scores at discharge: independent (FIM ≥6; n = 100) and dependent (FIM ≤5; n = 120). Logistic regression analysis assessed whether PhA at admission predicted ambulatory independence at discharge. Additionally, receiver-operating characteristic curve analysis determined optimal cutoff values.

**Results:**

Logistic regression analysis showed that PhA at admission and the National Institutes of Health Stroke Scale (NIHSS) were significant predictors of independent walking. The optimal cutoff values for PhA were determined to be 4.35° for men and 4.1° for women. Similarly, the cutoff scores for the NIHSS were 7.5 points for men and 5.5 points for women.

**Conclusions:**

In stroke patients, PhA and NIHSS at admission were significantly associated with ambulatory independence at discharge. Evaluation of PhA and NIHSS at admission may be useful for more accurate prediction of gait outcomes.

## Introduction

Walking ability is an important determinant of independence in performing activities of daily living (ADL)^1)^, and impaired walking ability is a major challenge following stroke^2)^. Therefore, it is essential for therapists to understand the factors influencing a patient’s ability to regain walking function poststroke. Previous studies have identified several factors associated with walking ability in poststroke patients, including age^3)^, cognitive function^4)^, lower limb muscle strength^4)^, lower limb motor paralysis^5)^, skeletal muscle mass^6)^, and trunk function^7)^. However, early in the post-hospitalization period, accurately assessing lower extremity muscle strength and trunk function may be challenging owing to the need for a stable sitting posture. Additionally, patients with cognitive impairments may struggle to follow instructions, further complicating accurate assessments at admission.

In contrast, bioelectrical impedance analysis (BIA) offers a noninvasive and straightforward method for evaluating skeletal muscle while the patient is in a supine position, making it widely used in clinical practice^8,9)^. BIA-derived skeletal muscle mass has been associated with both walking ability^6)^ and ADL performance^10)^ in stroke patients, making it a valuable predictor of functional prognosis.

The European Working Group on Sarcopenia updated its diagnostic criteria in 2019 to include skeletal muscle quality for sarcopenia^11)^. Studies in community-dwelling older adults have reported that muscle quality assessment is more strongly correlated with physical function than muscle mass alone^12,13)^, leading to increased attention on this measure. BIA-derived phase angle (PhA) is a surrogate marker of water distribution and somatic cell volume and has been recognized in clinical practice for assessing both nutritional status and muscle quality^14,15)^. A higher PhA indicates better cellular integrity and muscle quality^15)^. PhA has also been linked to sarcopenia diagnosis and improved ADL performance in stroke patients, suggesting its potential relevance in predicting functional outcomes^16,17)^. However, no previous studies have examined the association between PhA at admission and the recovery of gait independence poststroke. Elucidating this association could enhance prognostic accuracy for gait recovery and inform rehabilitation strategies.

Therefore, this study aimed to investigate the relationship between PhA at admission and gait independence in stroke patients. Additionally, we sought to establish a PhA cutoff value at admission to predict the likelihood of achieving gait independence.

## Methods

This retrospective observational study included stroke patients admitted to our recovery and rehabilitation unit after discharge from the acute care unit between April 2021 and December 2024. Eligible participants were stroke patients aged 65 years or older. Exclusion criteria included patients who had difficulty walking independently prior to stroke onset, those already able to walk independently at admission, those with severe cognitive impairment, individuals transferred back to the acute care unit, patients who died due to sudden deterioration, patients with paralysis on both sides of the body, those with implanted pacemakers, and cases with missing outcome data. Additionally, patients with metal implants were excluded, as orthopedic prostheses and implants may interfere with BIA results^18)^.

This study was reviewed and approved by the Ethical Review Committee of the Okayama Saiseikai General Hospital (ID: 250304). All experimental procedures were performed in accordance with the principles of the Declaration of Helsinki (revised October 2013).

### Data collection

Baseline patient information was collected within 48 hrs of admission. The collected data included age, sex, height, weight, body mass index, stroke subtype (ischemic or hemorrhagic), side of paralysis, Charlson Comorbidity Index (CCI)^19)^, National Institutes of Health Stroke Scale (NIHSS) score^20)^, time from stroke onset to hospitalization, length of hospital stay, pre-stroke ADL assessed using the modified Rankin Scale (mRS)^21)^, Cognitive function assessed using the Mini Mental State Examination Japanese (MMSE-J)^22)^, lower limb motor paralysis assessed using the Brunnstrom recovery stage (BRS)^23)^, functional independence Measure (FIM) mobility gait score^24)^, and average daily rehabilitation duration (min/day) during hospitalization. The CCI is an index of multimorbidity that scores 19 conditions related to chronic diseases, with higher scores indicating a greater number of comorbidities. The NIHSS is a standardized scale for evaluating stroke severity, comprising 11 items, including assessments of consciousness, motor function, sensory function, and speech, with each item rated on a scale from 0 to 4. Higher NIHSS scores indicate greater neurological impairment. The mRS is a commonly used clinical outcome measure to assess the degree of disability or dependence in ADL among individuals who have suffered a stroke or other neurological disorders. The scale ranges from 0 (no symptoms) to 6 (death), with higher scores indicating more severe disability. It is widely utilized in both clinical practice and research due to its simplicity and relevance to functional outcomes. The MMSE-J is an 11-item assessment tool of cognitive functioning. The higher the score, the better the cognitive function, with a minimum total score of 0 and a maximum of 30. The BRS categorizes motor recovery stages from I to VI, with higher scores indicating better motor recovery.

### Body composition

Skeletal muscle mass and PhA were assessed using BIA. Limb skeletal muscle mass was calculated as the sum of skeletal muscle mass in all 4 limbs, and the Appendicular Skeletal Muscle Mass Index (ASMI) was determined by dividing limb skeletal muscle mass by the square of height^25)^. PhA was calculated using the following equation: BIA-derived PhA = arctangent (Xc/R) × (180/π), where R represents the resistance and Xc represents the reactance measured at 50 kHz^18)^. Measurements were performed by a trained physical therapist using a body composition analyzer (InBody S10; Inbody, Tokyo, Japan) at the time of admission, after 15 min of rest in the supine position and at least 2 hrs after a meal.

### Primary outcome

The primary outcome was independent walking at discharge. Patients were classified as independently walking if their FIM mobility gait score was greater than 6, indicating that they could walk continuously for at least 50 m with or without an assistive device^7)^.

### Statistical analysis

The normal distribution of the data was assessed using the Shapiro–Wilk test. Categorical variables were reported as counts (percentages). Differences between the independent and nonindependent walking groups were analyzed using either an unpaired t-test or a Mann–Whitney U-test for continuous variables, and a chi-square test or Fisher’s exact test for categorical variables. Logistic regression analysis using the forced entry method was performed to evaluate the association between PhA and independent walking at discharge. Explanatory variables were selected from factors that showed significant differences in comparisons between the 2 groups. Multicollinearity was assessed using the variance inflation factor (VIF), with values below 5 confirming the absence of significant collinearity among variables. Pearson’s correlation coefficient was used to evaluate the relationship between ASMI and PhA, as previous studies have reported a correlation between the two^26)^. Multicollinearity was considered to be present when the correlation coefficient exceeded 0.6. The results of the multiple logistic regression analysis were reported as odds ratios (ORs) with 95% confidence intervals (CIs). Receiver-operating characteristic (ROC) curve analysis was used to plot sensitivity against 1 − specificity. The area under the curve (AUC) was calculated to determine the ability of factors at admission to identify independent ambulation at discharge. The Youden index was utilized to identify optimal cutoff values. Given that skeletal muscle mass and PhA are generally higher in men than in women, analyses were conducted separately by sex^17,27)^. Statistical significance was set at p <0.05, and all analyses were performed using the Modified R commander (version 4.4.1 for windows; Hirosaki Universitry, Aomori, Japan).

## Results

**Fig. 1.**
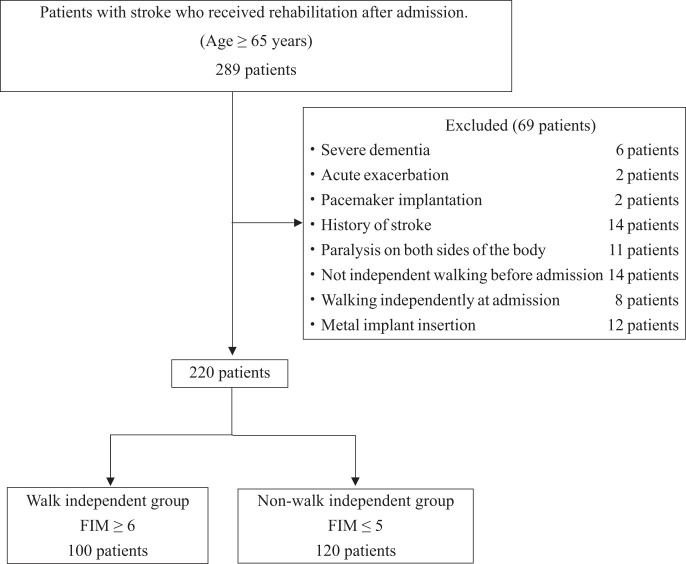
Flowchart of patient selection FIM, functional independence measure

A total of 289 stroke patients were initially screened for eligibility. After applying the exclusion criteria, 69 patients were excluded, resulting in a final sample of 220 patients (121 men and 99 women) included in the analysis. The mean age of the participants was 78.1 ± 9.3 years. Of these, 100 patients (54 men and 46 women) were classified into the walking-independent group, and 120 patients (67 men and 53 women) into the walking-nonindependent group (Fig. 1). Compared to the walking-nonindependent group, the walking-independent group was significantly younger and had significantly lower NIHSS scores. In contrast, scores for lower limb BRS, MMSE, ASMI, and PhA were significantly higher in the walking-independent group (Table 1). Pearson’s correlation analysis demonstrated a significant positive association between PhA and SMI in both men (r = 0.42, p <0.05) and women (r = 0.34, p <0.05). However, as the correlation coefficients did not exceed the threshold of 0.6, multicollinearity was not deemed present. To identify factors independently associated with walking independence at discharge, logistic regression analysis was conducted. The dependent variable was walking independence at discharge, and explanatory variables included age, MMSE-J, lower extremity BRS, ASMI, PhA, and NIHSS—variables that exhibited statistically significant differences between the walking-independent and walking-nonindependent groups. The results indicated that PhA and NIHSS were independently and significantly associated with walking independence in both sexes (Table 2). In men, the OR for PhA was 0.16 (95% CI: 0.02–0.27, p <0.01), while the OR for NIHSS was 1.62 (95% CI: 1.29–2.23, p <0.01). In women, the OR for PhA was 0.11 (95% CI: 0.01–0.29, p <0.01), and that for NIHSS it was 2.62 (95% CI: 1.8–4.62, p <0.01). ROC curve analysis further supported the predictive utility of PhA and NIHSS for walking independence at discharge. The AUC for PhA was 0.81 in men and 0.84 in women, while the AUC for NIHSS was 0.80 in men and 0.86 in women. The optimal cutoff value for PhA was determined to be 4.35° in men (sensitivity: 65.6%; specificity: 83.4%) and 4.1° in women (sensitivity: 77.3%; specificity: 83.4%). For NIHSS, the optimal cutoff value was 7.5 points in men (sensitivity: 77.3% specificity: 89.1%) and 5.5 points in women (sensitivity: 82.2%; specificity: 87.1%).

**Table 1.** Characteristics of patients in the walking-independent and walking-nonindependent groups

	All patients (n = 220)	Independent group (n = 100)	Nonindependent group (n = 120)	p-Value
Age (years [SD])	78.1 ± 9.3	76.3 ± 8.6	79.5 ± 9.5	<0.01
Sex (n (%))				0.78
Male	121 (55)	54 (54)	67 (55)	
Female	99 (45)	46 (46)	53 (45)	
Weight (kg [SD])	52.9 ± 10.8	55.7 ± 0.3	53.6 ± 10.7	0.09
Height (m [SD])	1.57 ± 0.1	1.57 ± 0.1	1.57 ± 0.1	0.98
BMI (kg/m^2^ [SD])	21.8 ± 3.3	22.3 ± 2.8	21.6 ± 3.4	0.51
Stroke type (n (%)				0.12
Cerebral infraction	149 (68)	73 (73)	76 (58)	
Cerebral hemorrhage	71 (32)	27 (27)	44 (42)	
Paralysis (n (%))				0.14
Right	113 (51)	46 (43)	67 (56)	
Left	107 (49)	54 (57)	53 (39)	
Onset-admission (days [SD])	21.1 ± 7	20.2 ± 6.1	22.1 ± 7	0.07
Length of hospital stay (days [SD])	85.2 ± 22.6	84.3 ± 16.4	85.7 ± 26.5	0.11
Premorbid mRS (points [IQR])	0 [0–1]	0 [0–1]	1 [0–2]	0.06
MMSE-J (points [SD])	19.9 ± 3.7	21.8 ± 3	18.3 ± 3.4	<0.01
Lower limb BRS (points [IQR])	4 [3–6]	4 [3–5]	4 [2–5]	0.02
CCI (points [IQR])	1 [1–2]	1 [0–2]	1 [1–2]	0.18
NIHSS (points [IQR])	7 [3–11]	4 [2–7]	10 [6–13]	<0.01
ASMI (kg/m^2^[SD])				
Male	6.2 ± 0.8	6.5 ± 0.7	6 ± 0.8	<0.01
Female	4.9 ± 0.7	5.2 ± 0.5	4.6 ± 0.8	<0.01
PhA (deg. [SD])				
Male	4.4 ± 0.7	4.8 ± 0.5	4.1 ± 0.7	<0.01
Female	4.2 ± 0.6	4.6 ± 0.4	3.9 ± 0.5	<0.01
FIM walk at admission (points [IQR])	2 [2–4]	2 [2–4]	2 [1–4]	0.07
Rehabilitation volume (min/day [SD])	114.5 ± 6.3	115.9 ± 7.1	113.8 ± 5.3	0.15

Measurements are represented as mean ± standard deviation [SD] or interquartile range [IQR], and number (percentage).

BMI, body mass index; mRS, modified Rankin scale; MMSE-J, Mini Mental State Examination-Japan; BRS, Brunnstrom recovery stage; CCI, Charlson comorbidity index; NIHSS, National Institutes of Health Stroke Scale; ASMI, appendicular skeletal muscle mass index; phA, phase angle; FIM, functional independence measure

**Table 2.** Logistic regression analysis of walking independently

Factor	Male (n = 121)			Female (n = 99)		
	Odds ratio (95% CI)	p-Value	VIF	Odds ratio (95% CI)	p-Value	VIF
Age	0.98 (0.91–1.06)	0.75	1.32	0.98 (0.89–1.06)	0.64	1.38
MMSE-J	0.94 (0.72–1.22)	0.67	1.04	0.97 (0.81–1.21)	0.82	1.18
Lower limb BRS	1.76 (0.73–4.41)	0.26	1.27	1.51 (0.66–4.05)	0.36	1.32
ASMI	0.56 (0.17–1.71)	0.31	1.32	0.84 (0.3–2.31)	0.08	1.11
phA	0.16 (0.02–0.27)	<0.01	1.24	0.11 (0.01–0.29)	<0.01	1.32
NIHSS	1.62 (1.29−2.23)	<0.01	1.16	2.62 (1.8−4.62)	<0.01	1.25

Male: χ^2^ test p <0.05, Hosmer–Lemeshow test p = 0.13. Female: χ^2^ test p <0.05, Hosmer–Lemeshow test p = 0.75.

CI, confidence interval; VIF, variance inflation factor; MMSE-J, Mini Mental State Examination-Japan; BRS, Brunnstrom recovery stage; ASMI, appendicular skeletal muscle mass index; phA, phase angle; NIHSS, National Institutes of Health Stroke Scale

## Discussion

The primary objective of this study was to examine the association between PhA, assessed using BIA, and the acquisition of walking independence in stroke patients undergoing rehabilitation. The results showed that PhA, along with NIHSS, was significantly associated with the achievement of walking independence and may play an important role in the rehabilitation process. Previous studies have shown an association between PhA and sarcopenia^16)^ or functional prognosis as measured by FIM^17)^ in stroke patients. However, these studies have all focused on the assessment of overall general condition and ADL, and have not specifically predicted gait function. The present study raises new clinical implications in that the outcome was gait independence at discharge from the hospital, and the predictive potential of this outcome was demonstrated by measuring PhA early in hospitalization.

Skeletal muscle mass, assessed using BIA, has previously been associated with walking ability^6)^ and ADL performance^10)^ in stroke patients. However, our logistic regression analysis demonstrated that PhA was significantly associated with walking independence, compared to ASMI, a conventional measure of skeletal muscle mass. Studies in community-dwelling elderly individuals have shown that muscle quality is more closely related to physical functions, including upper and lower limb strength, than skeletal muscle mass^12,13)^. Similarly, research in elderly patients with orthopedic conditions suggests that muscle quality deteriorates earlier than skeletal muscle mass^28)^. These findings suggest that assessing muscle quality, rather than skeletal muscle mass, may provide a better prediction of functional outcomes in the early stages of hospitalization. This study is the 1st to demonstrate that PhA is a more useful predictor of walking independence than ASMI in stroke patients undergoing rehabilitation.

The NIHSS was also selected as a significant predictor of walking independence in this study. The NIHSS is an index that reflects the neurological severity of stroke and has been reported to be associated with functional prognosis in stroke patients^29)^. In particular, the NIHSS is unique in that it can collectively assess various neurological symptoms that may indirectly affect walking ability, such as sensory function and attention functions, in addition to motor function of the lower limbs^20)^. In the present study, NIHSS was extracted as a predictor, while the lower limb BRS^4)^, which has been reported to be associated with walking prognosis in previous studies, was not selected as a significant factor. This result suggests that the NIHSS may have contributed to the prediction of walking independence in a more comprehensive manner, since it is not merely an index to assess motor paralysis, but reflects the entire neurological picture, including attention and higher brain function. Therefore, the NIHSS may be useful as an index that captures the multifaceted factors that may influence walking ability.

Furthermore, age^3)^ and cognitive function^4)^, which have been reported to be associated with walking in previous studies, were not identified as significant predictors of walking independence in this study. Regarding age, PhA is known to reflect age-related deterioration in muscle quality^30)^, and thus, the influence of aging may have been statistically accounted for through PhA. Additionally, the participants in this study were predominantly in their late 70s to 80s, resulting in a narrow distribution of age, which may have reduced the statistical power to detect its effect. With regard to cognitive function, participants were admitted to a convalescent rehabilitation ward after the acute phase, and patients with severe cognitive impairment had been excluded prior to enrollment. As a result, MMSE scores were concentrated in the moderate range, with mean scores of 21 and 18 in the independent walking group and nonindependent walking groups, respectively. This narrow range of scores may have reduced the likelihood of detecting a statistically significant association between cognitive function and walking independence. Furthermore, although the MMSE is useful for global cognitive screening, it has limited capacity to assess higher-order cognitive domains such as attention, executive function, and visuospatial abilities, which are more directly related to walking ability^31)^. Taken together, while cognitive function may have had some clinical relevance, its predictive value was relatively lower compared to PhA and NIHSS, and thus it was not selected as a significant predictor in the multivariate model.

The PhA cutoff values (4.35° for men, 4.1° for women) identified in ROC analysis had high specificity, suggesting that patients with PhA scores below these thresholds are unlikely to achieve independent ambulation. However, the sensitivity of these cutoff values was lower, meaning that some patients with PhA scores above these thresholds were still unable to walk independently. In addition to PhA, the NIHSS (cutoff scores of 7.5 for men and 5.5 for women) was selected as a predictor of walking independence in this study. Therefore, patients with a PhA score of 4.35° or higher (for men) or 4.1° or higher (for women), who have difficulty with gait independence should be evaluated for the NIHSS. The AUC values were high for both the PhA (0.81 for men and 0.84 for women) and the NIHSS (0.8 for men and 0.86 for women), suggesting that these ratings are strong predictors of gait recovery. PhA and the NIHSS reflect distinct aspects, namely, muscle quality and neurological function, respectively. Therefore, the combination of these 2 measures is considered to enhance the accuracy of predicting walking independence. Patients with scores above the identified thresholds may benefit from early rehabilitation goals and discharge planning, while those with lower scores may require more intensive rehabilitation strategies.

Resistance and aerobic exercise, as well as combining exercise with protein intake, have been reported to improve skeletal muscle quality^32–34)^. PhA, as a measure of muscle quality, has been shown to increase with exercise and decline with inactivity^35)^. This suggests that PhA may serve as an immediate marker of rehabilitation effectiveness. Based on our findings, patients with PhA scores <4.35° (men) and <4.1° (women) at admission may benefit from targeted interventions such as resistance training, aerobic exercise, and nutritional support, including protein supplementation, to improve muscle quality.

This study has certain limitations. First, the subjects in this study tended to have relatively mild motor paralysis, as indicated by the distribution of lower extremity BRS scores, and there were few cases of severe paralysis. Therefore, the results obtained in this study may not be reproducible in patients with more severe strokes. Future large-scale studies including more severe cases are needed. Second, lower extremity muscle strength and trunk function, which have been shown to affect walking ability after stroke, were not assessed in many patients and were not included in this study. Additionally, the nonindependent walking group in this study exhibited impaired cognitive function, suggesting that physical activity and skeletal muscle mass may have decreased prior to admission. Future studies should consider confounding factors such as physical activity, lower extremity muscle strength, and trunk function before admission to reduce selection bias. Finally, PhA and NIHSS, which were found to be associated with walking independence in this study, are only predictors and not factors that directly improve walking independence. Furthermore, this study was conducted at a single institution and needs to be validated for replication in different rehabilitation systems. Therefore, future intervention studies are needed to validate the clinical utility based on these measures.

## Conclusions

This study demonstrates that PhA and NIHSS at admission are significant predictors of ambulatory independence at discharge in stroke patients. Patients with PhA scores ≥4.35 (men) and ≥4.1 (women), and NIHSS scores ≥7.5 (men) and ≥5.5 (women) are more likely to regain independent ambulation, aiding early rehabilitation planning.
